# Introducing ActiveInference.jl: A Julia Library for Simulation and Parameter Estimation with Active Inference Models

**DOI:** 10.3390/e27010062

**Published:** 2025-01-12

**Authors:** Samuel William Nehrer, Jonathan Ehrenreich Laursen, Conor Heins, Karl Friston, Christoph Mathys, Peter Thestrup Waade

**Affiliations:** 1 School of Culture and Communication, Aarhus University, 8000 Aarhus, Denmark; 202204724@post.au.dk (S.W.N.); 202204836@post.au.dk (J.E.L.); 2 Department of Collective Behaviour, Max Planck Institute of Animal Behavior, D-78457 Konstanz, Germany; 3 VERSES Research Lab., Los Angeles, CA 90016, USA; k.friston@ucl.ac.uk; 4 Queen Square Institute of Neurology, University College London, London WC1N 3BG, UK; 5 Interacting Minds Centre, Aarhus University, 8000 Aarhus, Denmark; chmathys@cas.au.dk (C.M.); ptw@cas.au.dk (P.T.W.)

**Keywords:** active inference, free energy principle, predictive processing, Markov decision process, cognitive modelling, Julia, 87.15.Aa, 91-08, C63

## Abstract

We introduce a new software package for the Julia programming language, the library ActiveInference.jl. To make active inference agents with Partially Observable Markov Decision Process (POMDP) generative models available to the growing research community using Julia, we re-implemented the pymdp library for Python. ActiveInference.jl is compatible with cutting-edge Julia libraries designed for cognitive and behavioural modelling, as it is used in computational psychiatry, cognitive science and neuroscience. This means that POMDP active inference models can now be easily fit to empirically observed behaviour using sampling, as well as variational methods. In this article, we show how ActiveInference.jl makes building POMDP active inference models straightforward, and how it enables researchers to use them for simulation, as well as fitting them to data or performing a model comparison.

## 1. Introduction

We introduce a novel software library for Julia, ActiveInference, which lets users produce the simulated behaviour of agents and their internal belief states with active inference (AIF) models, as well as fit such models to empirically observed behaviour. AIF [1,2,3] is a generally applicable formal framework for understanding and simulating intelligent behaviour that is based in neurobiology and first principles from statistical physics [4,5,6,7,8]. AIF treats action and perception as unified under a joint imperative: to minimise the variational free energy (*VFE*), which quantifies how well the agent’s internal generative model explains incoming sensory observations. It is an upper bound on the the surprise from sensory observations, making AIF formally related to prediction error minimisation [9]. Choosing actions that minimise the expected free energy (*EFE*) of their consequences provides a natural balance between exploratory and exploitative behaviour; generalises descriptive approaches to behavioural modelling, like reinforcement learning and expected utility maximisation; and provides a singular approach to adaptive behaviour that can be used across different environments. AIF was argued to be applicable to any self-organising system that actively maintains a stable boundary that defines its integrity [10], a broad category that includes cells and plants [11], as well as humans [2] and even collectives [12]. Owing to its generality, AIF has seen a rise in popularity across multiple fields. It is used for theoretical simulations of the mechanisms underlying various types of behaviour [2], computational phenotyping in computational psychiatry [13,14], and agent-based simulations of population dynamics [15], as well as in engineering and robotics [16]. In AIF, perception and concurrent action are based on performing a variational Bayesian inversion of a generative model of the environment (i.e., a model of how the environment changes and brings about sensory observations). This belief updating includes inferring (hidden) states of the environment, learning parameters of the generative model and learning the structure of the generative model. Since the requisite inference schemes come pre-specified, the main task in AIF modelling becomes specifying an appropriate generative model. This includes specifying priors over environmental states, as well as what might be called *prior preferences*, *preference priors* or *goal priors*: immutable prior expectations that make up an agents’ preferences by furnishing a set of predictions over future states or observations; in fulfilling these predictions, free energy is minimised. The space of possible generative models is vast, and they often have to be handcrafted for a given environment. However, there are some families of generative models that can be considered “universal” in the sense that they can be used for most environments. Currently, the most popular of these is the discrete state-space Partially Observable Markov Decision Process (POMDP)-based generative models. Since they are ubiquitous in the literature, we focus here on making these types of generative models available to researchers. There are, however, other types of universal generative models, like generalised filtering models [17] or Hierarchical Gaussian Filtering-based models [18,19], that will be implemented in the future.

Tools for simulating POMDP-AIF models were originally developed as part of the DEM [20] library for Matlab [21] (part of the larger SPM library [22]). Since then, a modal and flexible software package pymdp [23] was created for Python [24], as well as a performance-oriented package cpp-AIF [25] for C++ [26] that can be used across platforms. Finally, the factor graph library RxInfer [27] for Julia [28] has also been used to implement some AIF models on an efficient factor graph back-end [29,30,31]. The important tools that these packages provide make AIF available for researchers to perform simulation studies and for use in engineering contexts. They do not, however, usually allow for fitting models to empirically observed data, which is a fundamental method used in cognitive modelling [32], often in the context of computational psychiatry [13], to infer the mechanisms underlying variations in behaviour or to investigate the differences between (for example, clinical) populations. Smith and colleagues [33] provided a guide for manually doing variational Bayesian parameter estimation based on empirical data, but only in Matlab and restricted to a particular class of variational parameter estimation methods (variational Laplace), instead of the sampling-based methods that currently predominate in the field of cognitive modelling [34,35].

In this paper, we introduce ActiveInference.jl, a new software library for Julia [28] that aims to provide easy-to-use tools for model fitting with AIF models and to introduce AIF to the growing community of researchers using Julia for computational psychiatry and cognitive modelling. Julia is a free and open-source high-level programming language that retains an easy user interface reminiscent of that in Matlab and Python. Simultaneously, Julia uses its “just-in-time” (JIT) compilations via the LLVM framework to approach the speed of languages like C without relying on external compilers [36]. Julia is also natively auto-differentiable, which means it can solve what is called the two-language problem (i.e., that high-level languages often have to rely on lower-level languages, either for performance or for auto-differentiability; this is the case with standard tools for cognitive modelling, where languages like R [37] must rely on external languages like STAN [38] for Bayesian model fitting). This means that ActiveInference, in conjunction with Turing [39], Julia’s powerful library for Bayesian model fitting, and its newly developed extension for behavioural modelling, ActionModels, makes it possible to use cutting-edge Markov Chain Monte Carlo [40] methods, as well as variational methods [35], for Bayesian model fitting with AIF. Crucially, this allows researchers to not only simulate AIF in a fast programming language, but to also fit them to empirical behaviour, as is performed in cognitive modelling and computational psychiatry. Importantly, this also places AIF models in an ecosystem of other models for computational psychiatry so that it can easily be compared with models, like Hierarchical Gaussian Filters [41], and reinforcement learning models, like the classic Rescorla–Wagner model [42]. As part of making ActiveInference.jl available to the scientific community, and to the larger software ecosystem within computational psychiatry, it is implemented as part of the Translational Algorithms for Psychiatry-Advancing Science (TAPAS) ecosystem [43].

In the next section, we provide a conceptual and formal introduction to AIF, particularly in the context of using POMDP generative models. In Section 3, we demonstrate how to use the package in practice, both for simulation and parameter estimation. In Section 4, we give a fully worked example of how ActiveInference can be used with a concrete simulated dataset. Finally, we discuss potential applications and future directions for developing the package.


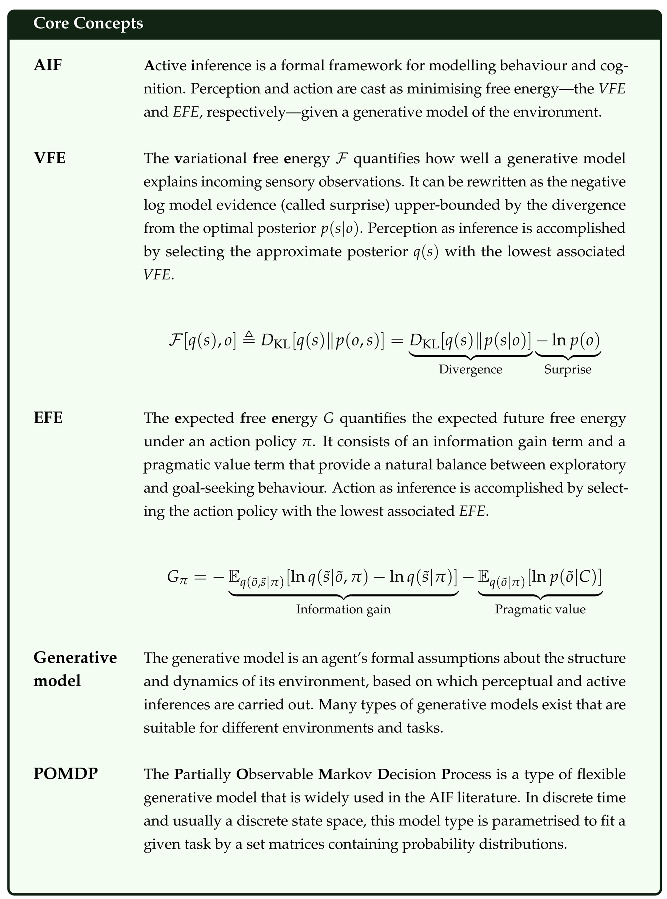


## 2. Active Inference with POMDPs

In this section, we briefly describe the core concepts of AIF and POMDPs. This should familiarise the reader with the vernacular used in the later sections regarding the functionalities of the package. While various extensions, such as structure learning, which enables an agent to learn the structure or shape of its environment through model comparison [44,45,46,47], or hierarchical and temporally deep POMDPs [48,49], are relevant for future work, describing these in detail is beyond the scope of this foundational paper.

At the core of AIF lies the minimisation of a variational free energy upper bound on surprise for perception, as well as action. This is motivated by the free energy principle [4,5,6,7,8], which states that self-organising systems can be described as minimising the variational free energy of their sensory states. The minimisation of free energy generally takes two quantities as its target: the variational free energy (*VFE*) in the case of perception and the expected free energy (*EFE*) in the case of action. The *VFE* is the free energy associated with a given sensory observation and is resolved perceptually by updating beliefs about the environment. The *EFE* is the free energy that is expected in the future, contingent on a given policy or course of action. Choosing action policies associated with a low *EFE* lead to reducing uncertainty about the environment, as well as making preferred observations more likely.

### 2.1. POMDPs in Active Inference

In AIF, the POMDP is one of the most common families of generative models used to make inferences about the environment. It is a Markovian discrete state-space model, where employing it means representing the environment and observations as inhabiting one among a set of possible (possibly multidimensional) states, and that the changes in these states can only depend on the system’s previous state and the agent’s actions. Environmental states are not directly observable, so they have to be inferred based on incoming sensory observations. In AIF for POMDPs and other generative models in general, both perception and action are cast as Bayesian inferences (see Section 2.2 and Section 2.3), as well as the learning of parameters of the generative model (see Section 2.4). Crucially, an agent’s generative model does not a priori have to be isomorphic to the true environment (i.e., the data-generating process), although this will generally lead to a successful inference, and that the generative model will therefore often come to resemble the environment through learning.

**Figure 1:**
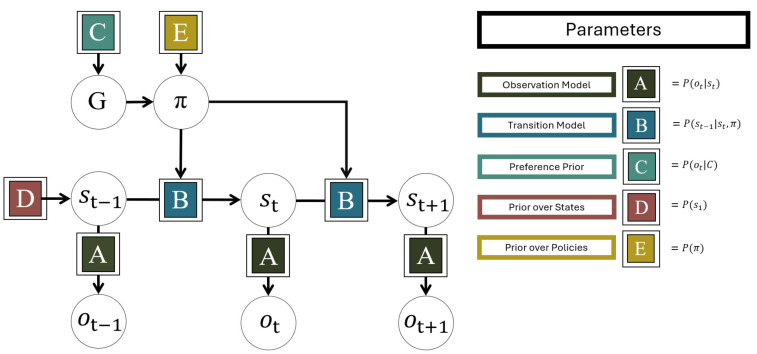
Depiction of a POMDP generative model. This encodes the agent’s expectations about how the state *s* of the environment changes over time *t*, and how it generates observation *o* at each time step. **A**, also called the observation model, describes how environmental states give rise to observations. **B**, also called the transition model, describes how environmental states change over time, depending on action *u* (called policy π when structured into sequences). **C** is the preference prior, which encodes the agent’s preferences for observations. This shapes the expected free energy *G* associated with each policy, which is used for policy selection. **D** encodes the agent’s prior belief over environmental states before making any observations, and **E** is the prior over policies that determines the agent’s preferences for policies in the absence of other motivation.

A discrete state-space POMDP in AIF is conventionally defined by five main sets of parameters: **A**, **B**, **C**, **D** and **E** [1,33], see Figure 1. Together, these parametrise the agent’s prior beliefs about the prior probability of different states in the environment, how states of the environment change and how they generate observations. Typically, they will be vectors, matrices or tensors; however, henceforth we denote them by their corresponding letter in bold. These make up the components needed for the agent to perform AIF.

**A**, also called the *observation model*, represents the state-to-observation likelihood model. This describes how observations depend on or are generated by states of the environment. It is structured as a matrix with a column for each possible environmental state *s*, and a row for each possible observation *o*. Each column is then a categorical probability distribution over the observations that will occur given the environmental state (meaning that each column must contain non-negative values that sum to 1). If the observations are multidimensional (i.e., multiple observations are made at each time point), there is a matrix for each observation modality. If two or more states determine the observation, the likelihood model then becomes a tensor. If **A** is imprecise (i.e., the probabilities are highly entropic and evenly distributed), observations are taken to carry less information about the environment, in many cases leading to more uncertain inferences, and vice versa.
**B**, also called the *transition model*, describes the state-to-state transition probabilities of environmental states *s*. **B** encodes the agent’s assumptions about how the environment changes over time, depending on its actions. It has a column and a row for each environmental state *s*, where each column is a categorical probability distribution over the states the environment will take on the next time step, given the state it is currently in. If the environment is modelled as multidimensional, there will be a matrix for each environmental state factor. Additionally, there is a separate matrix for each possible action (making each factor in **B** a tensor). This means that for every factor in the model, there may be one or more actions that pick out the appropriate slice of the tensor. Action therefore allows the agent to predict that the environment (and the corresponding observations) will change differently depending on the actions that it chooses. If **B** is imprecise (i.e., highly entropic), it means that the transitions of the environment are expected to be uncertain (and therefore, often transition to new states). In this sense, volatile and unstable environments will lead to less certain predictions about the future.
**C**, also called the *preference prior*, is a prior preference over possible observations. It encodes the types of observations that an agent a priori expects to encounter; since minimising expected free energy through AIF entails taking actions that make the predicted observations come about, **C** also encodes the agent’s preferences. It is a single categorical probability distribution over possible observations; if the observations are multidimensional, there is a separate *preference prior* for each observation modality. If **C** is imprecise (i.e., highly entropic), its preferences are weak and it will prioritise collecting information over realising its preferences; if it has low entropy, the agent will have stronger preferences and instead prioritise preferred outcomes or goals.
**D**, also called the *state prior*, is the agent’s prior belief about the states of the environment. It specifies the agent’s belief about the environmental state before receiving any observations. There is a separate *state prior* over environmental states for each factor. With a more precise **A**, the influence of the **D** quickly diminishes since the likelihood overwhelms the prior in the Bayesian inference.
**E**, also called the *habit prior*, is the prior over policies or paths. In the AIF vernacular, policies are allowable sequences of actions, with some specified policy length or temporal depth. **E** encodes the agent’s preferences for choosing certain policies in the absence of plans based upon *expected free energy*, sometimes called the agent’s “habits”. It is a single probability distribution over each possible policy.  

In addition to the five matrices, there are several hyper-parameters that are not part of the generative model, but are part of the inference algorithm. Here, we include two of the most common: the γ and α (inverse) temperature parameters. γ, the precision over policies, is the inverse temperature of a softmax transformation of expected free energies over policies, which is covered later in this section. After policies have been selected for a given time step, they are marginalised to calculate the probabilities of taking each possible action in the next time step. α, the action precision, is the inverse temperature of a softmax transformation on these final action probabilities, with higher values resulting in more stochastic action selection.

As noted, here we focus specifically on the POMDP-based generative models often used in the AIF literature. However, the basic steps when performing AIF—perception, action and learning—remain the same across generative models. In the remainder of this section, we describe each of these three steps in turn.

### 2.2. Perception in Active Inference

In AIF, perception is conceptualised as the result of variational (i.e., approximate) Bayesian inference, performed by minimising the *VFE* to optimise parameters of posterior beliefs about the environment. In exact Bayesian inference, we use a parametrised generative model *m* to make an optimal inference about state *s* of the environment based on observation *o*. This is performed by combining a prior belief over states p(s|m); a likelihood model p(o|s,m); and the model evidence p(o|m), a normalisation term encoding the likelihood of receiving the given observations across all possible environmental states, as follows [1]:

(1) $$p\left(s|o,m\right)=\frac{p(o|s,m)p(s|m)}{p(o|m)}$$

The posterior distribution over states given observations p(s|o,m) here represent the agent’s beliefs about the environment. Forming beliefs in this way is thought to be the process that enables conscious, as well as unconscious, perception. The product of the likelihood model and prior is also called the joint likelihood p(o,s|m), which fully defines the generative model, and which we use henceforth. In the following, for notational simplicity, we also omit denoting the dependency on the generative model *m*.

Calculating the model evidence p(o) is often intractable, making exact Bayesian inference unfeasible. The way to circumvent this in AIF is to use a variational approximation to Bayesian inference [23,33,50,51]. This works by transforming the inference into an optimisation problem, specifically the minimisation of the *VFE*. First, an arbitrary probability distribution over environmental states q(s), an approximate posterior that is used to approximate the exact posterior, is introduced. We then introduce the Kullback–Leibler (KL) divergence between the approximate posterior q(s) and the exact posterior, which is also sometimes called the perceptual divergence:

(2) $$D_{\mathrm{KL}}\left[q\left(s\right)∥p\left(s|o\right)\right]=\sum_{s}q\left(s\right)\ln\frac{q(s)}{p(s|o)}$$

It is a property of the KL divergence that the two distributions are identical when $D_{\mathrm{KL}}\left[q\left(s\right)∥p\left(s|o\right)\right]=0$. Minimising this divergence then corresponds to approximating the exact posterior p(s|o) with q(s). We cannot evaluate this divergence directly since the exact posterior is still unknown. We therefore replace the expression of the exact posterior with the right-hand side of Equation (1). Note that here we use the joint likelihood p(o,s) notation, fraction rule $\frac{a}{\frac{b}{c}}=\frac{a}{b}*c$ and logarithmic rule ln(a∗b)=lna+lnb:

(3) $$\sum_{s}q\left(s\right)\ln\frac{q(s)}{\frac{p(o,s)}{p(o)}}=\sum_{s}q\left(s\right)\ln\frac{q(s)}{p(o,s)}+\ln p\left(o\right)$$

We can now rewrite the first term of the right-hand side as the KL divergence of the approximate posterior from the joint likelihood, which is equal to the expression used in Equation (2):

(4) $$D_{\mathrm{KL}}\left[q\left(s\right)∥p\left(s|o\right)\right]=D_{\mathrm{KL}}\left[q\left(s\right)∥p\left(o,s\right)\right]+\ln p\left(o\right)$$

We now define the *VFE* (F[q(s),o]) as the KL divergence of the approximate posterior from the joint likelihood. The VFE is only a function of q(s) and *o* (and the generative model *m*), and we can therefore calculate it without knowing the model evidence p(o):

(5) $$F≜D_{\mathrm{KL}}\left[q\left(s\right)∥p\left(o,s\right)\right]=\sum_{s}q\left(s\right)\ln\frac{q(s)}{p(o,s)}$$

The probability-weighted sum can be rewritten as an expectation, and the joint likelihood can be decomposed into a prior and a likelihood:

(6) $$F≜E_{q(s)}\left[\ln\frac{q(s)}{p(o,s)}\right]=E_{q(s)}\left[\ln q(s)-\ln p(o|s)-\ln p(s)\right]$$

We can now combine our definition of *VFE* with Equation (4):

(7) $$D_{\mathrm{KL}}\left[q\left(s\right)∥p\left(s|o\right)\right]=F\left[q\left(s\right),o\right]+\ln p\left(o\right)$$

Finally, we can reorganise this equation to show that the VFE is the sum of the divergence of the approximation posterior and exact posterior (if we could perform exact inference, this is what we would obtain) and the surprise ℑ=−ln(p(o)) (the negative log model evidence):

(8) $$F\left[q\left(s\right),o\right]=\underset{\mathrm{Divergence}}{\underset{︸}{D_{\mathrm{KL}}\left[q\left(s\right)∥p\left(s|o\right)\right]}}\underset{\mathrm{Surprise}}{\underset{︸}{-\ln p(o)}}$$

Since the KL divergence is non-negative, the *VFE* becomes an upper bound on the surprise:

(9) F[q(s),o]≥−lnp(o)

By rearranging the parts of this expression, we can express the *VFE* as a balance between the complexity and accuracy, where the accuracy is how well the model predicts observation, and the complexity is how much the beliefs need to change in order to maintain a high accuracy:

(10) $$F\left[q\left(s\right),o\right]=\underset{\mathrm{Complexity}}{\underset{︸}{D_{\mathrm{KL}}\left[q\left(s\right)∥p\left(s\right)\right]}}-\underset{\mathrm{Accuracy}}{\underset{︸}{E_{q(s)}\left[\ln p\left(o|s\right)\right]}}$$

Since the *VFE* can be calculated (Equation (5)), it can be used as a target for minimisation. This allows us to choose the approximate posterior q(s) that is associated with the smallest *VFE*; since the surprise does not depend on q(s), minimising the *VFE* this way must necessarily reduce the divergence between the approximate and exact posterior. We now, as is usually performed in variational inference, introduce a mean-field approximation [35] so that the joint approximate posterior $q(s_{t})$ factorises across time steps t∈T and hidden state factors f∈F:

(11) $$q\left(s\right)=q\left(s_{1},s_{2},\ldots ,s_{T}\right)=\prod_{t=1}^{T}q\left(s_{t}\right)$$

(12) $$q\left(s_{t}\right)=q\left(s_{t}^{1},s_{t}^{2},\ldots ,s_{t}^{F}\right)=\prod_{f=1}^{F}q\left(s_{t}^{f}\right)$$

The factorisation into hidden state factors allows us to calculate the VFE separately for each factor *f* and sum them to obtain the total VFE. The factorisation in time allows us to calculate the time-specific VFE as in Equation (6) using the predictive posterior $\ln p(s_{t}|s_{t-1},u_{t-1})$ from the last time step as the prior:

(13) $$F_{t}=E_{q(s_{t})}\left[\ln q\left(s_{t}\right)-\ln p\left(o_{t}|s_{t}\right)-\ln p\left(s_{t}|s_{t-1},u_{t-1}\right)\right]$$

which is the value we intend to minimise. Various methods exist for minimising the *VFE*; the one used in pymdp, from which we drew much of our inspiration, is Coordinate Ascent Variational Inference (CAVI) [35], where the fixed points of the *VFE* are solved for with coordinate descent (also known as fixed-point iteration (FPI) [23]). This is also the algorithm currently available in ActiveInference.jl. We correspondingly use a coordinate descent update to find the factorised approximate posterior $q(s_{t}^{f})$ that minimises the time-dependent VFE $F_{t}$, and therefore optimises for the time-specific variational posterior $q(s_{t})$. To obtain the coordinate descent update, we start by taking the derivative of $F_{t}$ with respect to $q(s_{t}^{f})$ and setting the derivative to zero [23]:

(14) $$\frac{\partial F_{t}}{\partial q(s_{t}^{f})}=\ln q\left(s_{t}^{f}\right)+1-E_{q^{i\setminus f}}\left[\ln P(o_{t}|s_{t})\right]-\ln\left(E_{P(s_{t-1}^{f},u_{t-1}^{f})}\left[P(s_{t}^{f}|s_{t-1}^{f},u_{t-1}^{f})\right]\right)=0$$

Solving for $q(s_{t}^{f})$ yields

(15) $$\ln q\left(s_{t}^{f}\right)=E_{q^{i\setminus f}}\left[\ln P(o_{t}|s_{t})\right]+\ln\left(E_{P(s_{t-1}^{f},u_{t-1}^{f})}\left[P(s_{t}^{f}|s_{t-1}^{f},u_{t-1}^{f})\right]\right)-1$$

which leads us to the coordinate descent update equation:

(16) $$q^{*}\left(s_{t}^{f}\right)=\sigma \left(E_{q^{i\setminus f}}\left[\ln P(o_{t}|s_{t})\right]+\ln\left(E_{P(s_{t-1}^{f},u_{t-1}^{f})}\left[P(s_{t}^{f}|s_{t-1}^{f},u_{t-1}^{f})\right]\right)\right)$$

where $E_{q^{i\setminus f}}$ denotes the expectation over q(s) for factor *i*, where the posterior over states in the other factors *f* are kept constant. By iteratively solving (16), the FPI scheme will eventually find a local optimum and converge to a solution for the variational posterior. By default, ActiveInference uses 10 iterations or stops when $\partial F_{t}<0.001$. This posterior then comprises the AIF agent’s belief about the state of the environment, and therefore, its perception.

### 2.3. Action in Active Inference

As with perception, action in AIF is guided by the minimisation of free energy. However, instead of *VFE* being minimised directly, it is the free energy that is expected to occur depending on the actions taken by the agent—the expected free energy or *EFE*—that is minimised. As stated below, choosing actions that minimise the *EFE* leads to a natural balance between exploration and exploitation, ensuring preferences are realised and ambiguity about the environment is minimised. In AIF, policies π are sequences of actions *u*. The policy length (also called the planning horizon or temporal depth) is the length of the policies being considered. The total number of policies therefore depends on the policy length and the number of different actions that can be made at each time step. An *EFE* is assigned to each policy π (denoted as $G_{\pi }$), where policies associated with a lower *EFE* are then more likely to be chosen.

One can rewrite the *EFE* in different ways to highlight different consequences of optimising it. Below, we show the two most crucial ways to rewrite it, taken from [1,33]. We denote the states and observations that are expected future outcomes of actions with (~). Additionally, we introduce a *preference prior* **C** that encodes the agent’s preferences:

(17) $$G_{\pi }=-\underset{\mathrm{Information}\;\mathrm{gain}}{\underset{︸}{E_{q(\tilde{o},\tilde{s}|\pi )}\left[\ln q\left(\tilde{s}|\tilde{o},\pi \right)-\ln q\left(\tilde{s}|\pi \right)\right]}}-\underset{\mathrm{Pragmatic}\;\mathrm{value}}{\underset{︸}{E_{q(\tilde{o}|\pi )}\left[\ln p\left(\tilde{o}|C\right)\right]}}$$

The expression above shows how minimising the *EFE* leads to a natural balance between information gathering and realising preferences. The first term on the right-hand side is the change in belief from the prior to the posterior under a given policy called the epistemic value or information gain. Optimising this value is what leads to (notably non-random) exploratory behaviour. The second term is the pragmatic value; minimising this value ensures that observations are in accordance with the preference prior **C**.

Another way to express the *EFE* is in terms of risk and ambiguity:

(18) $$G_{\pi }=\underset{\mathrm{Expected}\;\mathrm{ambiguity}}{\underset{︸}{E_{q(\tilde{s}|\pi )}\left[H\left(p(\tilde{o}|\tilde{s})\right)\right]}}+\underset{\mathrm{Risk}\;\left(\mathrm{outcomes}\right)}{\underset{︸}{D_{KL}\left[q\left(\tilde{o}|\pi \right)∥p\left(\tilde{o}|C\right)\right]}}$$

Here, the first term on the right-hand side captures the expected entropy, or uncertainty, of the outcomes given the environmental states. Minimising this quantity ensures that the agent will seek states where observations can most clearly be used to distinguish between environmental states. The second term is the KL divergence of the expected observations from preferred observations, capturing the risk of making unwanted (i.e., a priori surprising) observations, which is also minimised by minimising the *EFE*.

### 2.4. Learning in Active Inference

In AIF, the parameters of the generative model can also be updated via Bayesian-belief-updating methods, a process called “parameter learning” or sometimes just “learning” [2]. In general, this is performed by introducing belief distributions over the possible values of the parameters that are subject to learning, and updating this distribution for each observation using Bayesian belief updating. This additionally implies introducing priors on the belief distributions. Depending on the type of generative model used, the belief distributions and their priors will take different forms, and so will their update equations. In the following, we demonstrate parameter learning specifically in the context of POMDPs.

The parameters that are subject to learning in POMDPs are usually the entries in the five matrices. Since the matrices consist of categorical probability distributions, it is natural to use Dirichlet distributions—distributions over categorical probability distributions—as belief distributions over their values [33,52]. Beliefs about each probability distribution Θ is then described by a Dirichlet distribution parametrised by a set of concentration parameters θ:

(19) p(Θ)=Dir(Θ|θ)

The concentration parameter of a Dirichlet distribution is essentially a non-negative count of how many times the given category (be it a type of observation or state transition) has occurred. The distribution of concentration parameter counts will determine the shape of the estimated categorical probability distribution, while the scale of the concentration parameters will determine the certainty per precision of the belief. Updating beliefs about Θ (the parameters in the matrices) then corresponds to updating these concentration parameters θ with the following update equation:

(20) $$\theta_{t+1}=\omega *\theta_{t}+\eta *\chi_{t}$$

The updated value for the concentration parameter $\left(\theta_{t+1}\right)$ is found by adding the previous concentration parameter $\theta_{t}$ multiplied by a forgetting rate ω to the observed data count χ (either the observation in the case of **A** learning, or the inferred state or state transition for other matrices) multiplied by a learning rate η. With this relatively simple update equation—which, in essence, amounts to just counting the occurrences of categories—an AIF agent can update its beliefs about the various matrices it uses to make inferences about environmental states. For more details on parameter learning with POMDPs, see [23,33,52].

## 3. Using ActiveInference.jl

In this section, we provide an overview of the various functions a user will need to operate ActiveInference. This includes functionalities for creating POMDP agents, for simulating behaviour and for fitting the models to data. In the next section, we demonstrate how to use the package on a concrete worked example. ActiveInference is under continual development, and the newest version of the package, including documentation for how to use it, can be found at github.com/ilabcode/ActiveInference.jl.

### 3.1. Creating and Using a POMDP

The general structure of ActiveInference.jl is heavily inspired by pymdp [23], a Python library for implementing simulations of AIF in discrete state spaces. Those already acquainted with pymdp should find the syntax here familiar. ActiveInference can be installed as normal from the official Julia General Registry using the Julia’s native package manager Pkg:


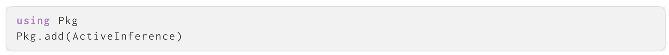


It can then be loaded into the current project environment:


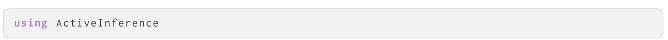


Central to the package is the AIF object. This is a structure containing all the components of the generative model, as well as the dynamic belief states and the various settings needed to perform AIF, and is used in conjunction with most of the high-level functions of the package. An AIF object can be created with the init_aif function, which takes as arguments the components of the generative model and a dictionary of various settings and parameters:


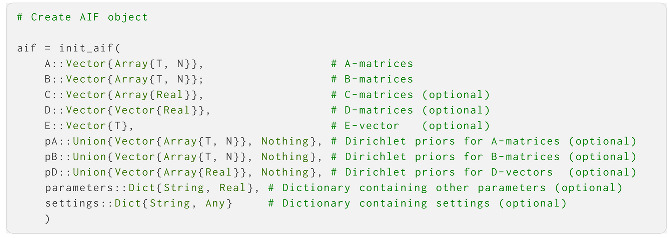


**A** and **B** are the only mandatory arguments to the init_aif function—the other arguments are keyword arguments that default to uniform priors. **A**, **B**, **C**, **D** and **E** and their corresponding Dirichlet priors, in the cases of **A**, **B** and **D**, should be formatted as standard array objects. All but **E** can have multiple modalities/factors (see Section 4), so they should be formatted as vectors of arrays with one array per modality/factor. These arrays can be hand-specified by the user, or be generated with some of the helper functions supplied by ActiveInference. Here, we create an AIF agent equipped with a generative model with six environmental states, five possible observations and two possible actions. Here, we use helper functions to create matrices and vectors with the correct dimensions; in Section 4, we create them manually. First, we define the number of states, observations, controls and the length of policies:


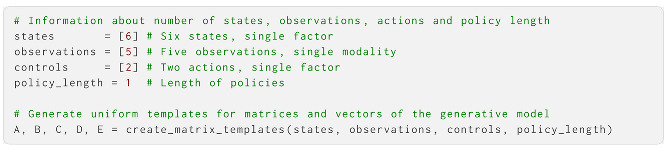


The **A** object generated here is a one-dimensional vector containing a uniform 5 × 6 matrix (six states and five observations). The **B** object is a one-dimensional vector containing a uniform 6 × 6 × 2 array (six states and two actions). The **C**, **D** and **E** objects are one-dimensional vectors, each containing uniform vectors with their corresponding sizes. We can now modify these to supply the agent with more informative priors over observations, initial states and policies. Here, we performed this using the onehot function:


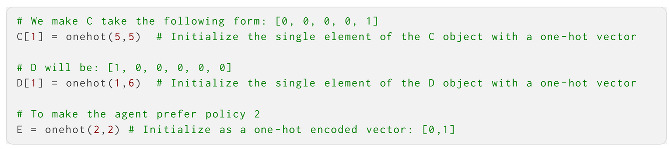


We now create the Dirichlet priors for **A**, **B** and **D**. When we use parameter learning, these are used to define **A**, **B** and **D** defined above, and are updated at every time step. One way to construct Dirichlet priors is to simply multiply the matrices below with a scaling factor; a higher scaling leads to more precise priors that require stronger evidence to update. Here, we use a scaling parameter of 2. In the current version, parameter learning is only implemented for the **A**, **B** and **D**:


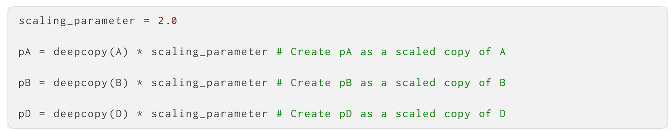


Finally, we define the hyper-parameters and other settings in two dictionaries. Here, we display the most common parameters and settings, and set them to their default values, which are used if they are not set by the user:


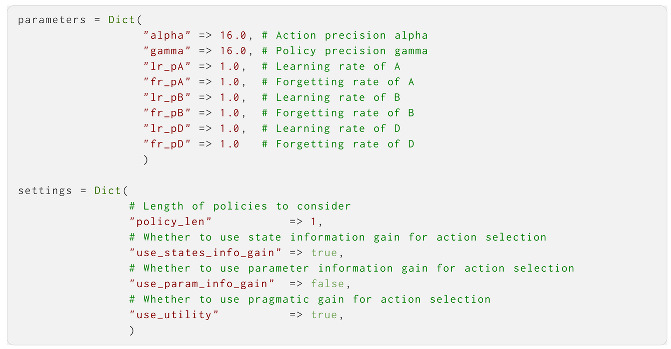


We can now input the above arguments into init_aif and create an AIF object. This allows us to present it with observations and let it choose actions. The following displays a few functions in the order they are usually used, first inferring environmental states, then updating matrices and then selecting a new action. First, we make inferences about the environment with infer_states!, which returns the updated posterior belief q(s) about environmental states given an observation. The observation is presented as a vector with an entry for each observation modality:


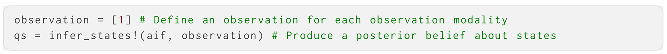


We can now also update the Dirichlet beliefs about the various parameters with the update_parameters! function. **A** is updated based on the last observation and the posterior belief about the state that generated it. **B** is updated based on the posteriors about the previous state transition given the previous action. **D** is updated based on the posterior over states at the first time step.


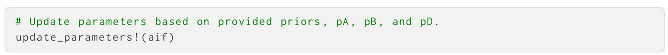


The infer_policies! function then calculates the expected free energy for each possible policy (the number of policies varies depending on the amount of possible actions per time step and the length of policies considered), as well as the corresponding posterior probability over these policies. This calculation depends on the settings specified in the init_aif function, including the policy length and which parts of the information gain to use, as well as the policy precision γ.


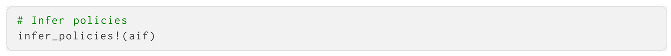


Finally, sample_action! then samples the next action from the agent. This is performed by marginalising the policy probabilities to obtain the probabilities for the action on the next time step, and then softmax transforming it with the α action precision parameter.


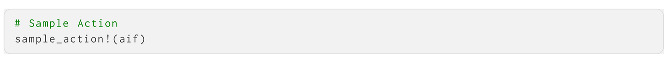


These functions can be combined by users in various ways, depending on their purpose. Often, however, users will want to combine them in a single function that implements the full action–perception loop that receives an observation and returns an action. This is implemented with the ActionModels sister package for behavioural modelling.

### 3.2. Simulation with *ActionModels*

ActionModels is a library for implementing, simulating and fitting various behavioural models to data. Here, we show how to use it in conjunction with ActiveInference to make the simulation of AIF models easy and in a fully generalised framework that is compatible with other types of cognitive and behavioural models as well. ActiveInference provides a full “action model”—a full model of the action-generating process in an agent—for using AIF called action_pomdp!. In this case, all this information is contained in the AIF object. action_pomdp! then takes the AIF object and a single-time-step observation as arguments, and then runs state inference, parameter learning and policy inference, and returns probability distributions over the possible actions of the agent.


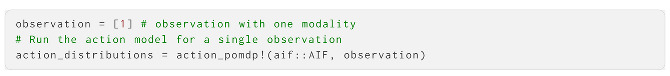


This can conveniently be used in conjunction with an ActionModels agent, a more abstract structure that is used for running behavioural models in general, and which is used when fitting models to data. We therefore begin with initialising an agent that contains the AIF object:


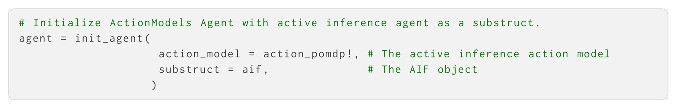


The agent object can be used with a set of standard functions. single_input! provides the agent with an observation, updates it is beliefs and returns a sampled action; for non-action-dependent observations, give_inputs! provides a series of observations across time steps and returns actions for each. These can be easily used in an agent-based simulation to have AIF agents evolve and act over time.


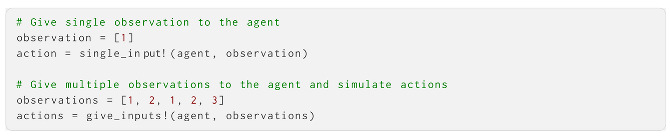


Additionally, a set of convenience functions can extract and set parameters and (histories of) beliefs. We briefly show how to extract the current or histories of past states:


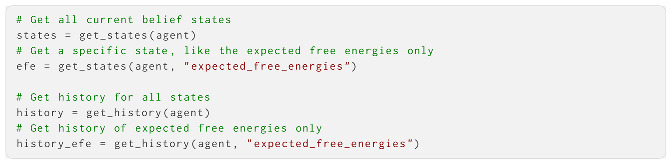


And how to change the parameters of a created agent:


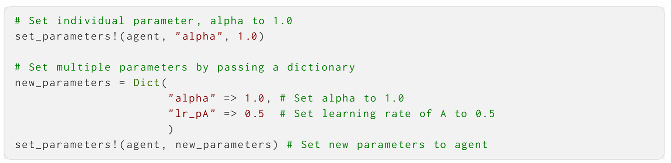


### 3.3. Model Fitting with *ActionModels*

In addition to simulating the behaviour and belief updating of agents, ActionModels also makes it possible to fit models to data and perform parameter estimation. This is used in general to form better models and theories of mental processes, as well as to find mechanistic differences (usually prior beliefs in AIF) between, for example, clinical populations or investigating how computational constructs, like Bayesian beliefs, relate to, for example, neuronal dynamics. This is performed in fields like cognitive modelling and mathematical psychology [34], as well as computational psychiatry [14,53]. In the following, we briefly describe the high-level functions needed to fit AIF models to empirical data with ActionModels.

We have our agent object defined as above. We then need to specify priors for the parameters we want to estimate. Here, we estimate the α parameter, and use a gamma distribution as prior:


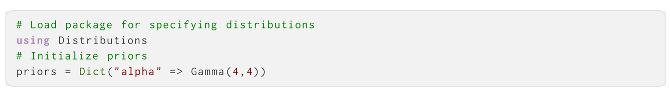


We can now use the create_model function to instantiate a probabilistic model object with data. This takes the agent object, the priors, and a set of observations and actions as the arguments:


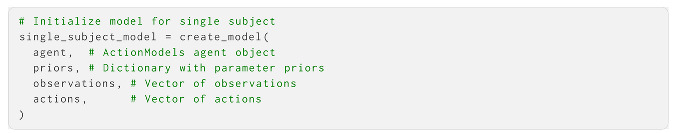


If we have multiple subjects whose parameters we wish to estimate, we can achieve this by passing a dataframe object to the same function. Here, we specify which columns are actions and inputs, as well as which column to use for grouping the specific time series:


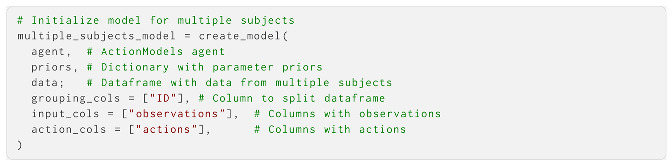


This model can be used as a normal Turing model object. ActionModels provides a convenience function for doing this with appropriate defaults:


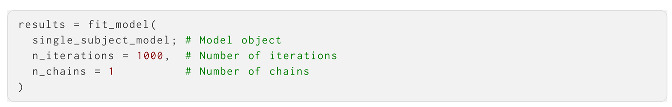


The output of the fit_model function is an object containing the standard Turing chains, which we can use to access the chain statistics:


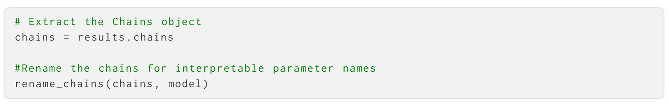


ActionModels provides a range of convenience functions for behavioural modelling. We can extract the posterior parameter estimates for each participant, and extract it in a convenient data frame structure for later processing:


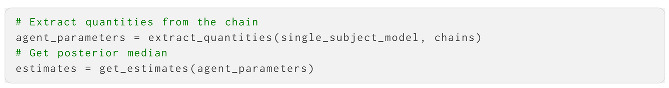


We can also sample parameter values from the prior and plot the posteriors against the priors:


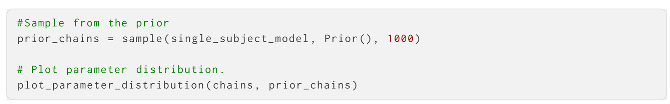


See the documentation for ActionModels at github.com/ilabcode/ActionModels.jl for various other functionalities, including modelling how parameters vary across a population, parameter recovery and predictive checks. In this section, we outline how to use ActiveInference for simulation and model fitting in conjunction with ActionModels. In the following section, we show how to achieve this on a concrete worked example.

## 4. Usage Example

In this section, we demonstrate a full usage example of how to create an AIF agent, simulate behaviour in a classic T-maze environment and fit the AIF agent to a simulated example dataset. We provide the necessary code to run this simulation. All code required to reproduce the example simulation can be found in an open source OSF repository osf.io/j3k5q/. This example was performed with the current version of ActiveInference.jl (0.1.1); the newest version can be found at github.com/ilabcode/ActiveInference.jl.

### 4.1. Setting Up Environment and Agent

A T-maze is a simple task commonly employed in the behavioural sciences, as well as in the AIF literature [14,54,55,56,57]. It is a minimal type of task that requires balancing exploration and exploitation, or epistemic and pragmatic value, respectively. It is also suitably represents in a discrete state space. Together, this makes it easily compatible with a POMDP-based AIF approach.

**Figure 2:**
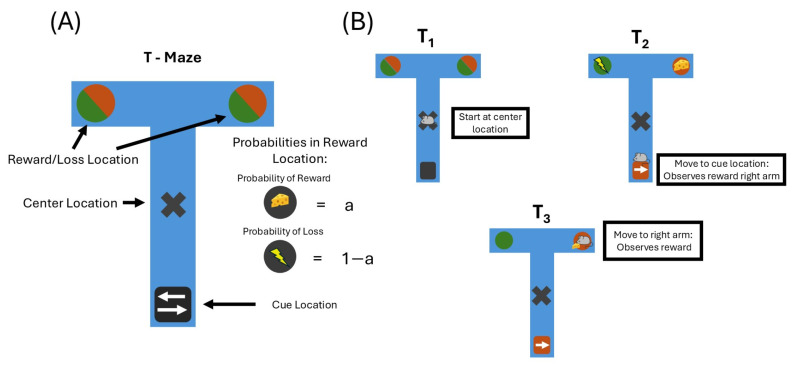
A depiction of the T-maze. (**A**) The full layout of the T-maze task, with the centre location, the cue location and the two reward conditions. (**B**) A three-step example of a T-maze trial. The agent (in this case, a mouse) starts at the centre location. In order to reduce the uncertainty regarding which arm the reward is located in, the agent moves to the cue location. The cue location reveals the right arm to be the reward location, and in the subsequent time step, it goes to the right arm and observes the reward with some probability.

The structure of the T-maze is, as the name suggests, a T-shaped maze, consisting of a centre location, a cue location (bottom of the T), and reward and loss locations (one in each arm of the T) (Figure 2). On every trial, the agent can move to one of the two arms of the T to receive a reward; one, called the reward location, will yield rewards with a higher probability than the other side. At the cue location, which the agent can move to, the agent receives a cue that indicates which of the locations is the reward location. Generally, the cue may be more or less informative; in this example, it always accurately reflected the reward conditions state (reward in the right or left arm). The reward location only provides a reward probabilistically. This means the agent can either take a chance and go directly to one of the two upper arms, or spend its first move seeking information about where the reward is before moving to the reward location. Since the clue location is not preferred, the second option comes with a cost in terms of pragmatic value, which has to be outweighed by the epistemic value in resolving uncertainty about the reward location state. Note that for the agent to realise that this uncertainty reduction will aid it in its subsequent choice of arm, it would have to be able to anticipate the effect of its actions on its own future beliefs, a process called “sophisticated inference” [58].

In the following, we proceed to describe the generative model used by the agent, which here was identically structured to the *generative process*, the actual environmental process generating the data. In the maze task, the environmental states and concurrent observations were multidimensional, while the actions were unidimensional. Here, we refer to the different dimensions of the environment as *state factors* and the different dimensions of the observations as *observation modalities*. There were two state factors, the location of the agent and the reward condition:
  **State Factor 1 (Location):**
Centre location;Right arm;Left arm;Cue location.
  **State Factor 2 (Reward Condition):**
Reward condition right;Reward condition left.


The first factor described which of the four locations in the maze the agent was inhabiting—the centre, cue location, and left and right arms—and was controlled by the action. The second factor was the reward condition factor, and had two possible states: the reward location being on the right or on the left. The agent’s actions directly controlled the first factor, while it had to infer the second based on the cue in order to complete the task. There were three observation modalities: one for observing the agent’s own location, one for observing rewards and one for observing the cue:
  **Modality 1 (Location):**
Centre location;Right arm;Left arm;Cue location.
  **Modality 2 (Reward):**
No reward;Reward;Loss.
  **Modality 3 (Cue):**
Cue right;Cue left.


The first observation modality had an observation for each of the positions in the maze, and generally reflected the agent’s actual position perfectly. The second modality was the agent’s observation of a reward or loss, which depended on the reward condition and the position. The “no reward” observation was received whenever the agent was not occupying either of the arm locations, where it did not observe any reward or loss. The third modality was the observation of the cue, which depended on the reward modality so that the agent could use it to infer the correct reward condition. The action was one-dimensional with four options and was used to move between the different locations:
  **Actions:**
Move to centre location;Move to right arm;Move to left arm;Move to cue location.


We now provide an example of using the package with a T-maze environment. First, we installed and loaded the package:


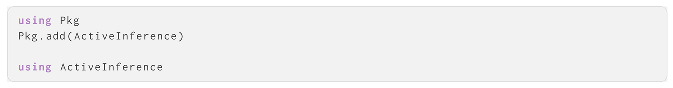


We first set up the environment or generative process: the T-maze. The T-maze is included as a pre-made environment in ActiveInference, so we simply loaded it. We set the reward probability for the reward condition to 95 percent:


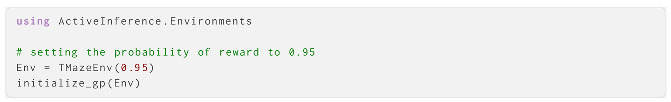


We then proceeded to set up **A**, **B**, **C**, **D** and **E**. For this we used the create_matrix_templates helper function to set up the correct structure and then populate it. To start, we defined what went into the helper function. It took five arguments: the numbers of states and observations in each factor and modality; the policy length; the number of controls; and lastly, what to initially populate them with. The specific states and observations are made clear below when discussing populating the parameters.

The first three arguments should be specified as vectors of integers, containing the numbers of states and observations for each factor or modality. In our case, we had two factors: a location factor and a reward condition factor, which had four and two states, respectively. There were three modalities: one location modality with four observations, one reward modality with three observations and one cue modality with two observations. Finally, there were four possible actions for controlling the location factor, and only one possible action for the reward state factor. The policy length was specified as an integer, in our case 2, and we populated the template with zeros.


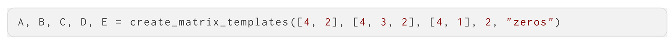


We started by defining **A**, or the observation model. In this example, since we allowed for **A** learning, **A** was not used directly, but we still defined it in order to construct the Dirichlet prior over it (and it could be used directly if **A** learning was not required). Of the three observation modalities, the first was the location observation. Here, there were four possible observations, mapped to the four location states and two reward condition states. This resulted in an **A** that was four location observations by four location states by two reward conditions, i.e., a 4 × 4 × 2 tensor. We let the agent correctly assume perfect observations of the location by specifying an identity matrix for **A** in each reward condition:


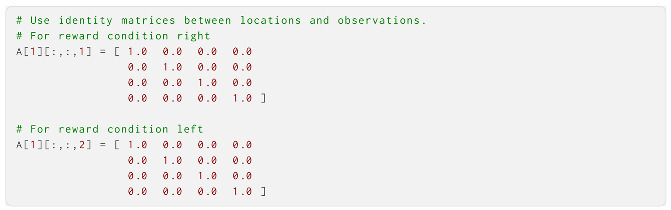


**Figure 3:**
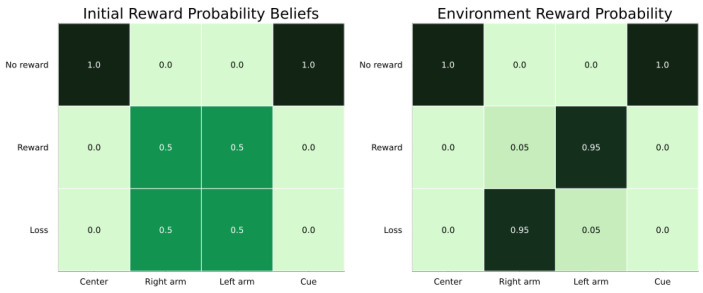
Reward probabilities for the four locations. The centre (column 1) and cue (column 4) locations always resulted in the “no reward” observation (row 1). The two arms (columns 3 and 4) resulted in either rewards (row 2) or losses (row 3), with some probability. Left: the agent’s agnostic starting beliefs about reward probabilities. Right: the true reward probabilities for the reward condition left arm, which the agent needed to learn over time. The amount of saturation of the green color represent the likelihood of a specific observation in a give state.

The second modality was the reward modality, which mapped the observations “no reward”, “reward” and “loss” onto the location states and reward conditions. For the second modality, we therefore had a tensor that was three reward observations by four location states by two reward conditions, i.e., a 3 × 4 × 2 tensor. When the agent was at the centre and cue locations, we let the modality accurately expect the observation of “no reward” with certainty. For the two arm locations, we let the agent be agnostic regarding whether they provided rewards or losses. This was different from the true reward probabilities (see Figure 3), which the agent needed to learn over time. This was the case for both reward conditions.


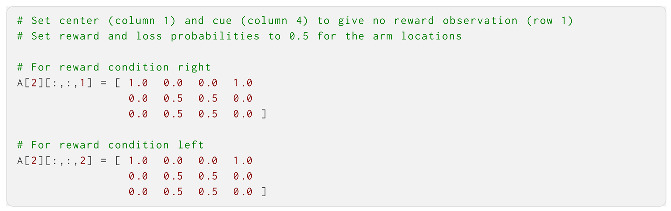


The third and last modality was the cue modality, which mapped the cue observations onto the location and reward condition states. This resulted in an **A** that was two cue observations by four location states by two reward conditions, i.e., a 2 × 4 × 2 tensor. The observations in this modality were correctly assumed by the agent to truthfully reveal the current reward condition—i.e., whether the right or left arm was better—when standing at the cue location. We implemented this by giving each cue observation equal probabilities at all locations except the cue location, where there was a perfect correspondence between the reward condition and the observation:


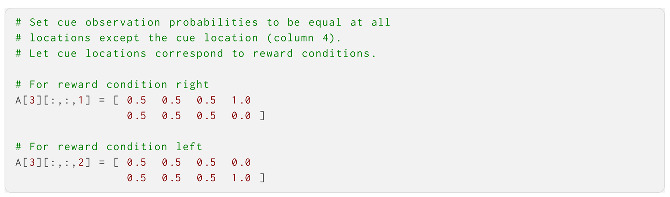


Having created all three modalities of **A**, we could continue to **B**, or the transition model. Each of the two state factors of **B**—the location factor and reward condition factor—needed to be defined separately. We started with the location factor, which contained the transition to and from four possible location states under four different actions: a 4 × 4 × 4 tensor. The agent could control these states perfectly with its four movement actions, independently of its current position. This was implemented by letting the probability of transitioning to a location be 1 for the location corresponding to the action chosen:


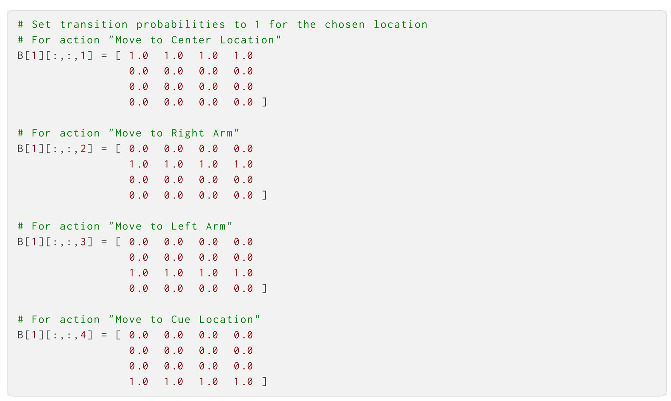


The reward condition factor in **B** here was rather simple, as we let the agent correctly assume that the reward condition never changed (although one could have enabled **B** learning to let the agent learn the transition probabilities of the environment). There were two possible states for these state factors, so we implemented this as a 2 × 2 identity matrix:


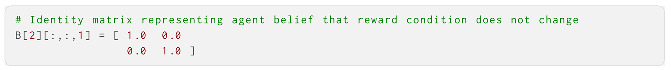


Then, we built **C**, the prior preference over observations. **C** encoded preferences for each observation in each modality: the location, reward and cue. The relative value of the entry corresponding to a specific observation defined the preference; here, we set all values with neutral preferences as 0, and used positive values for preferences and negative values for aversions. The only observations over which the agent had preferences were in the reward modality, where the rewards (index 2) were preferred and losses (index 3) were disliked. The strength of the preference determined the explore–exploit balance of the agent; here, we use 3 as the value:


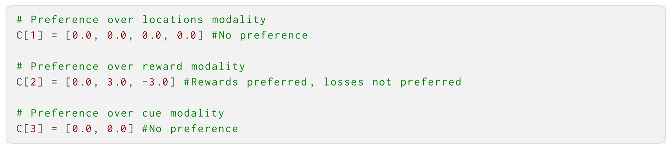


Then, we built **D**, which was the prior or initial belief over the environmental states. There was a vector in **D** for each of the two state factors: the location state and the reward condition. We let the agent start with a belief that it was at the centre location (index 1), and gave uniform priors for the reward condition:


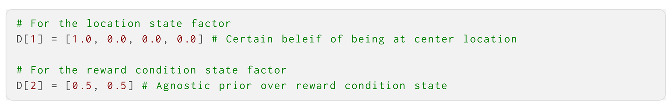


Finally, we established **E**, the prior over policies. This was a vector over all possible policies; with four possible actions and a policy length of 2, there were $4^{2}=16$ policies in total. Here, we gave the agent an agnostic prior over policies:


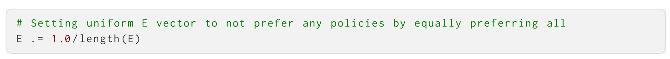


Thus, the five matrices were defined (we note that the C, D and E matrices were set to agnostic by default if not defined by the user). All that was left was to set the Dirichlet prior for **A** learning, and set the parameters and settings. In the following, we set the the Dirichlet prior for **A** to a scaled copy of the original **A** using a weak scaling parameter of 2.0:


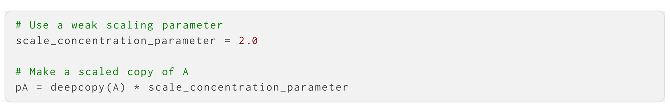


We then set the various hyper-parameters for the agent’s inference algorithm. Here, we used standard default values: an **A** learning rate of 1, and γ and α values of 16:


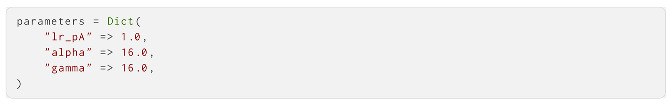


Finally, we defined the settings of the agent (other characteristics of the agent that were not estimable parameters). Here, we set the policy length to 2, which let the agent use expected information gain for both the state and parameter info gain for its actions, and specify that it was specifically the second modality of **A** that was to be learned:


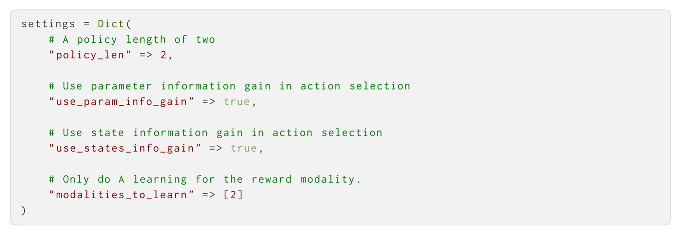


Having built all the necessary components of the generative model, we could then instantiate an AIF agent:


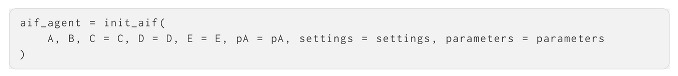


### 4.2. Simulating Behaviour

Since the environment and agent were set up, we could proceed to simulate the behaviour of the agent in the environment. We created a for loop, where the agent received an observation, made inferences about the environment, updated **A**, inferred policies and sampled actions:


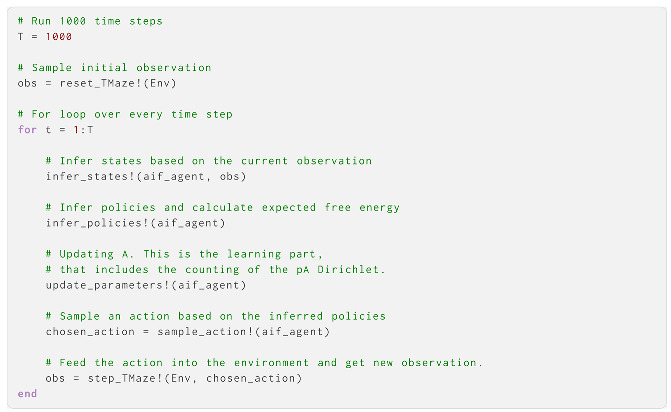


**Figure 4:**
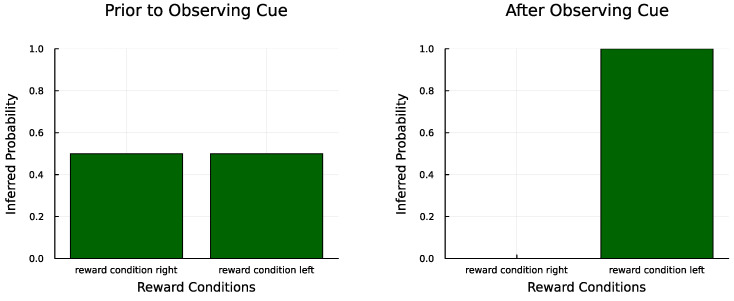
State inference for the reward condition. The agent’s beliefs about the reward condition changed from agnostic (**left**) to certain that it was the left reward condition (**right**) after observing the cue.

**Figure 5:**
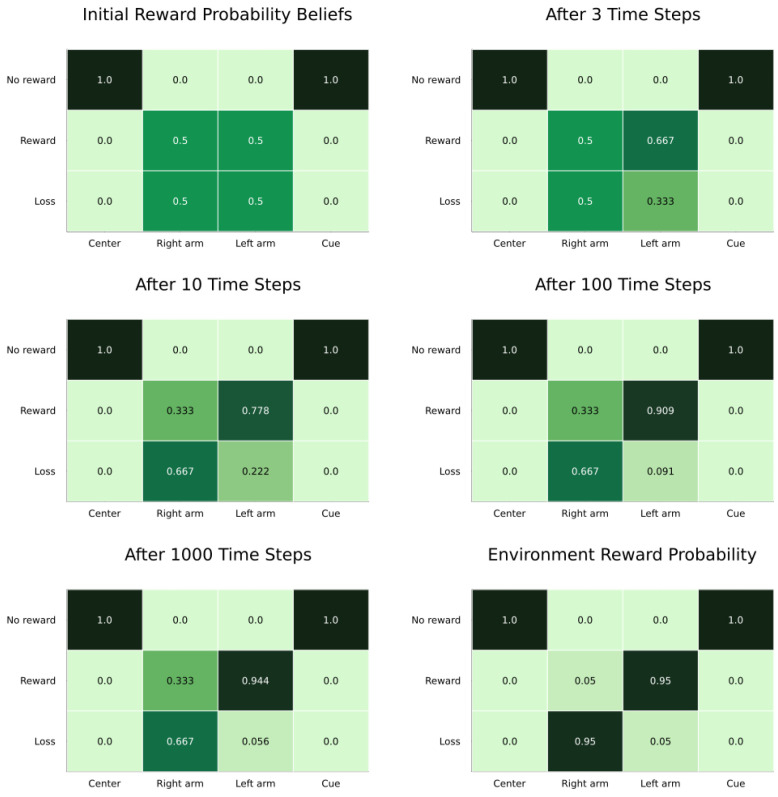
**A** learning for the actual reward condition (reward condition left). The agent correctly learned the probability of receiving rewards in the rewarding arm. It did not learn the probabilities of the non-rewarding arm since it did not explore that option. The color grading signifies the likelihood of an observation being generated by a specific state. The more saturated the color, the higher the likelihood.

The agent here started by moving to the cue location, and then proceeded to move to the left arm repeatedly. The main objects of learning here were the reward condition state and the **A** parameters for rewards under the two reward conditions. After observing the cue, the agent updated its belief (correctly) to be certain of being in the left reward condition (Figure 4). Over time, the agent learned the correct probability of receiving rewards in the left arm (0.94 versus a correct 0.95). It did not learn the probabilities for the right arm; this was because it never moved to that location, having already learnt that the left arm was more likely to produce rewards (Figure 5). This would be less likely to be the case with lower γ and α values, as well as a more entropic **C**.

### 4.3. Fitting the Model to the Data

Simulations are useful for a variety of purposes, like exploring the consequences of different priors and parameters and establishing the face validity of hypothetical mechanisms underlying behavioural phenomena. However, we often want to use models to make inferences about specific observed phenomena, like the differences in behaviour between various populations, as in computational psychiatry [14]. One standard method here is model fitting, where we estimate the parameter values (e.g., prior beliefs) of an AIF model that are the most likely given some observed behaviour of a participant. This is often performed with approximate Bayesian methods. In the cognitive and behavioural sciences, the predominant method is Markov Chain Monte Carlo (MCMC) methods [34], which are slower but in the limit can estimate parameter posteriors without making assumptions about their functional form. An alternative, which is more often used in other fields and also available in ActiveInference is variational methods, which are faster but require making assumptions about the functional form of the posterior. In general, MCMC methods are favourable when making parameter inferences (i.e., comparing parameters of the same model fitted to different data, like two groups of subjects). When performing a Bayesian model comparison (i.e., comparing different models fitted to the same data), the different approaches rely on different approximations of the model evidence, with the variational free energy having some claims to being a better approximation than the information criteria classically used with MCMC methods (although see other approximations, like the Pareto-Smoothed Importance Sampling [59] or Thermodynamic Integration methods [60]; see [35] for a further review). Note that independently of which of these approaches one might take, the process involves inverting a generative model of the mental processes underlying the behaviour of a given subject, a generative model which itself is an inversion of the subject’s generative model of the environment. We can call the generative model that the agent has of its environment the *subjective* generative model, and the model we have of the agent the *objective* generative model, in what has been called a meta-Bayesian approach or “observing the observer” [1,61].

Here, we demonstrated model fitting by fitting the POMDP model to the synthetic behaviour that it generated; this is called a parameter recovery study since we can then compare the estimated parameters to the generative values used for creating the simulated data [62,63]. Here, we used the simulation method shown in the previous section to produce a synthetic dataset with known parameter values for each agent (in practice, these are often participants in an experiment), here with a focus on estimating the α parameter. We then used MCMC methods to estimate the parameters for each agent and compared the estimated values with the correct values. Here, we simulated two groups of five synthetic subjects agents with different α values (the parameters for the first group were sampled from a Gaussian distribution with mean = 8 and SD = 2, and the second group with with mean = 24 and SD = 2). Each agent interacted with the T-maze environment for 300 time steps. We produced the following data frame, containing the data of each of the agents: their observations, actions and an identifier, a format suitable for cognitive and behavioural modelling.


3000×5 DataFrame

Row

Location

Int64

Reward

Int64

Cue

Int64

Action_Location

Int64

Action_Reward

Int64

SubjectID

Int64

1

1

1

1

4

1

1

2

4

1

2

3

1

1

3

3

3

2

2

1

1

.

.

.

.

.

.

.

.

.

.

.

.

.

.

3000

2

2

2

2

1

10


We used ActionModels to fit the AIF model created above to each of the agents in the dataset. We began by initialising an ActionModels agent:


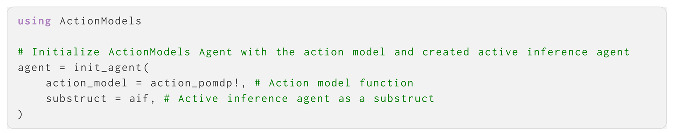


We then set the prior for the parameter we wanted to estimate: the α action precision. As an example, we chose a wide, weakly informative prior: a Gaussian distribution with mean 5 and standard deviation 5, truncated at 0 and 20:


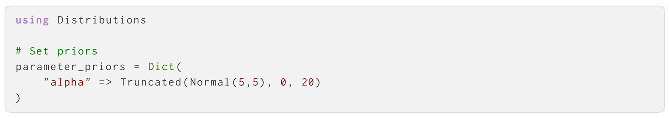


Next, we instantiated the probabilistic model with data and parameter priors:


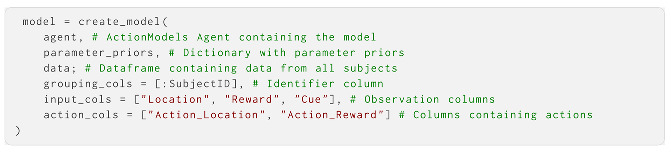


Finally, we used the fit_model function to perform (parallelised) parameter estimation for each of the agents:


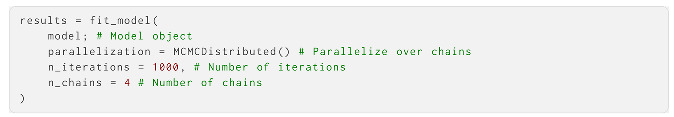


The output contained the chains object with the resulting posterior samples:


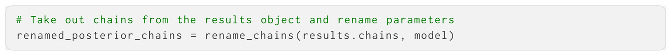



Summary Statistics

parameters

mean

std

mcse

ess_bulk

ess_tail

rhat

SubjectID:1.alpha

3.8785

0.2350

0.0034

4826.0114

2512.806

1.0016

SubjectID:2.alpha

2.9718

0.1945

0.0029

4523.9650

2781.4532

1.0033

SubjectID:3.alpha

3.3598

0.2147

0.0031

4816.1661

3054.7500

1.0016

.

.

.

.

.

.

.

.

.

.

.

.

.

.

SubjectID:10.alpha

8.9233

0.9660

0.0170

3551.1005

2126.9604

1.0013


**Figure 6:**
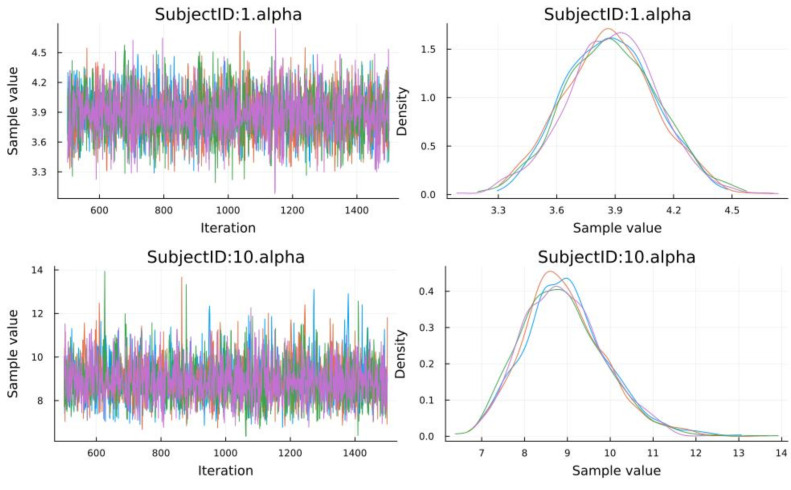
Chain traces. Each color signifies an individual chain.

We could plot the posteriors and chains, which is often performed to diagnose whether the sampling was successful (Figure 6):


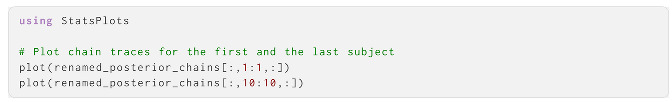


**Figure 7:**
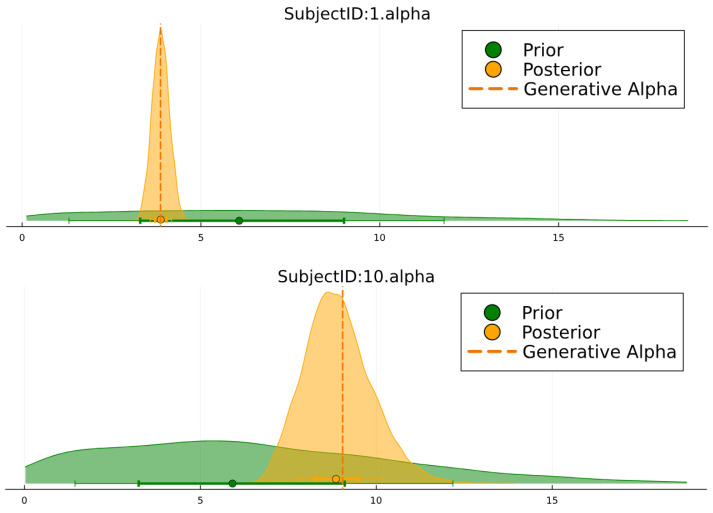
Posterior estimates of the α parameter plotted against the prior for two synthetic subjects, one from each group.

Further, we used plot_parameters from ActionModels to plot the posterior estimates against their priors. Here, we performed this for one agent from each group, also highlighting the parameter value used to generate the behaviour (Figure 7). We saw that the posterior was correctly centred around the generative value.


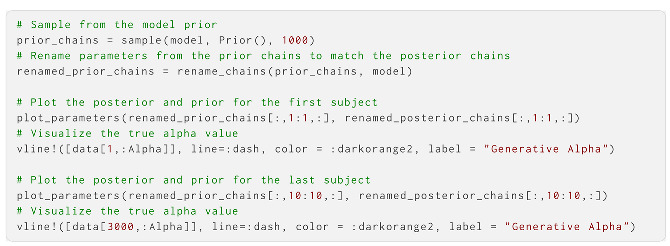


**Figure 8:**
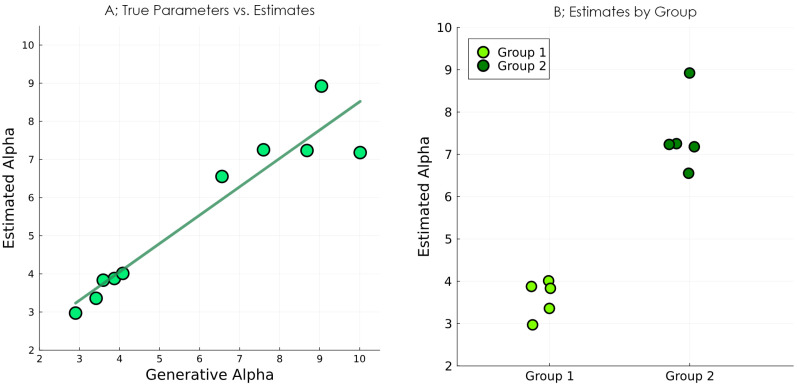
Results of the parameter recovery study. (**A**) Estimated parameter values plotted against the values used to generate the data. (**B**) Parameter estimates split by the two groups from which the parameter values of synthetic subjects were sampled.

We then, as is often the case in computational psychiatry, wanted to compare the distributions of parameter values between the two groups. We extracted the median of the estimated posteriors for each subject and plotted them against the value used to generate the behaviour (Figure 8). We saw that the estimation successfully captured the difference between the two groups, and that the α parameter recovered fairly well. Note that the ability to recover parameters depends on the specific model and task, as well as on the specific values of the parameters (when α is very high, for example, the behaviour becomes essentially deterministic; further increases in α would then not have any effect on the behaviour, and therefore, not be estimable). A subtle issue here is that the parameters that best explain some data are not necessarily the parameters used to generate those data. This is because the best parameters are those that maximise the marginal likelihood of the data (also known as the model evidence); because the model evidence includes a complexity term, the parameter recovery will often recover parameters that provide a simpler explanation for the data relative to the parameters used to generate these data.


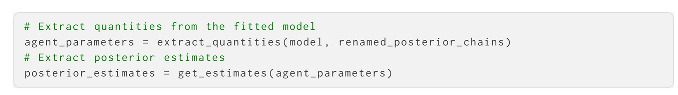


Finally, there are various metrics for model comparison that might be calculated, as implemented by various software packages. Here for demonstration, we calculated the Pareto-Smoothed Importance Sampling approximation to Leave One Out cross-validation (PSIS-LOO) [59], as implemented by ParetoSmooth.jl [64]:


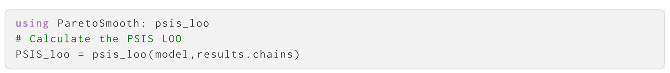


## 5. Discussion

We introduce ActiveInference.jl, a novel Julia software package for creating and using POMDP-based AIF models for simulation and fitting to empirical data, demonstrating its ease of use on a small parameter study with simulated agents. ActiveInference.jl makes AIF modelling available in a fast language, equipped with an interface and situated in an ecosystem oriented specifically towards cognitive and behavioural modelling.

Importantly, the ability to fit models to empirical data with sampling-based methods provides value to researchers within cognitive modelling and computational psychiatry: it allows for comparing estimated parameter values between population groups or investigating the temporal dynamics of belief changes in experimental participants. Dynamic belief trajectories can then be related to other (for example, physiological) measures, as is usual in model-based neuroscience [65]. This method can also, in principle, be used for fitting models to other types of experimentally observable systems, like animals, organoids [66], and simulated or emergent systems [67]. The package can also be used for agent-based modelling in general, for repeating earlier analyses with sampling based model-fitting and for comparing POMDP-based AIF models directly to other types of models.

Since they implement full approximate Bayesian inferences, AIF models are computationally more demanding than many approaches traditionally used in cognitive and agent-based modelling, in particular when the dimensionality of the generative model is large. This means that models with highly multidimensional or complex behaviour and large numbers of agents can be computationally infeasible to implement, especially given the additional computational demands introduced by fitting these models to empirical data. Avenues for addressing this implicit scaling problem were proposed in the context of machine learning applications [68,69], and with the use of simplifying assumptions—the use of which are ubiquitous in computational modelling—AIF has been used to model multi-agent phenomena, such as opinion dynamics [15,70], coordinated foraging [71] and fish school movements [12]. It remains to be explored how AIF models can be applied to highly complex natural phenomena, such as a concrete election, which underscores the need for efficient but flexible and accessible software tools in the field.

There are many ways in which ActiveInference can be improved. It would be useful to extend the set of dynamic belief states to include prediction errors since they are often used for model-based neuroscience. This would entail departing from discrete state-space (i.e., POMDP) models to consider continuous state-space models apt for Bayesian filtering or predictive coding (see below). An alternative would be to generate prediction errors from belief updating under discrete models, where prediction errors can be read as the (KL) divergence between posterior and prior beliefs (i.e., complexity or information gain). A simple interface could be added for creating custom parametrisations of the requisite parameters that could be parametrised with Boltzmann or Gibbs distributions, as opposed to Dirichlet distributions. Parameter learning could be extended to all generative model parameters, as well as in parametrised forms (e.g., so that the Boltzmann parameter or temperature of the parameters that are learned); similarly for the precision over expected free energies γ. Preference priors should also be implementable for environmental states, in addition to observations, and **A** can be made action dependent.

A library of pre-made canonical POMDP models could be created so that users can easily implement them directly. Alternatives to the fixed-point iteration method for updating posteriors over environmental states could be included, like the marginal message passing algorithm. There are various ways in which the package can be made more computationally efficient, and it could be compared with other software implementations. There are plenty of utility and plotting functions that could be added to the package to make it easier to use and to facilitate integration with the model-fitting packages it relies on; for example, to allow for combining the models with linear regressions to compare parameters values of different populations in a single model. More complex types of POMDP models can also be added, like hierarchical and temporally deep POMDPs. Model structure learning could be considered, where different model structures are compared and chosen between by evaluating their free energies. Sophisticated inference, where predictions are also made about changes in one’s own beliefs—depending on expected action-dependent observations in the future—could also be implemented [58]. Finally, the package could be extended to other types of generative models than POMDPs, including other universal models, like generalised filtering [17] and Hierarchical Gaussian Filter models [41], as well as custom generative models, or even (deep learning-based) amortised inference models. These various extensions could provide valuable tools for using AIF models in both theoretical and applied research.

## Data Availability

The original data presented in this study are openly available in ActiveInferenceJuliaPaper at URL: https://osf.io/j3k5q/.

## Abbreviations

The following abbreviations are used in this manuscript:

AIF	Active inference
FEP	Free energy principle
VFE	Variational free energy
EFE	Expected free energy
MCMC	Markov Chain Monte Carlo
POMDP	Partially Observed Markov Decision Process
